# Experimental study on soil-water characteristics and strength characteristics of complete-intense weathering mudstone in seasonal frozen area

**DOI:** 10.1371/journal.pone.0285484

**Published:** 2023-05-15

**Authors:** Haotian Guo, Runjian Zhou, Chao Sun, Yuli Lin, Zhe Wang

**Affiliations:** School of Geometrics and Prospecting Engineering, Jilin Jianzhu University, Changchun, Jilin, P.R. China; University of Vigo, SPAIN

## Abstract

To study the soil-water characteristics and shear strength of unsaturated complete-intense weathering mudstone, the soil-water characteristic curve of complete-intense weathering mudstone and the matric suction of mudstone samples under natural state were measured. This measurement was through a soil-water characteristic test using a pressure plate instrument. Based on the results of soil-water characteristic test, an unsaturated (saturated) triaxial shear test was carried out. Under different temperatures and confining pressure conditions, the shear strength and deformation characteristics of complete-intense weathering mudstone under natural water content and saturation conditions were investigated. The results show that the percentage of silt and clay in unsaturated complete-intense weathering mudstone in natural state is relatively high, with the mudstone having less sand, and a weak permeability and exhibiting a significant capillary phenomenon. The complete-intense weathering mudstone with a natural moisture content of 19.15% has a matric suction of 210 kPa. When the temperature is constant, the shear stress of the sample increases with the increase of confining pressure. When the temperature decreases from 0 to -20°C, the influence of confining pressure on the rock samples gradually decreases. The rock sample has the property of strain hardening during shearing. Under the same matric suction, the total cohesion increases with the decrease of temperature. At a positive temperature, the effective internal friction angle increases with the decrease of temperature. At a negative temperature, the lower the temperature, the smaller the effective internal friction angle. The test of shear strength parameters of saturated complete-intense weathering mudstone is simple and conservative. In practical engineering, the basic properties of unsaturated complete-intense weathering mudstone can be predicted by testing the shear strength parameters of saturated complete-intense weathering mudstone. The results of the study are important for better understanding the nature of unsaturated complete-intense weathering mudstone and improving the safety of engineering construction in complete-intense weathering mudstone areas.

## 1 Introduction

In 2022, China, Russia, and Mongolia confirmed the extension of the "Outline for the Construction of China-Mongolia-Russia Economic Corridor". So, the new construction and reconstruction of underground structures in many cities in this corridor will be carried out in full swing, and complete-intense weathering mudstone is often encountered in the depth range of these structures. Some constructions such as foundation pit excavation, tunnel construction, and subway construction need to be completed in winter. Complex hydrological, geological and temperature conditions directly affect the safety and investment of underground structures. Therefore, it is of practical significance for underground engineering construction to study the soil-water characteristics of complete-intense weathering mudstone and the influence of different temperatures on the strength and deformation characteristics of unsaturated and saturated complete-intense weathering mudstones.

Unsaturated soil is composed of solid, liquid, and gaseous phases. In the seepage analysis of unsaturated soil, the water storage capacity of soil is not found a constant, but is related to the change of suction. Also, soil-water characteristic curve (SWCC) is a function of water content and suction [1,2]. Based on the soil-water characteristic curve, the permeability coefficient of unsaturated soil can be calculated [3] and the strength of the soil can be speculated [4]. Numerous scholars have gone forward and backward in the study of soil-water characteristic curve of unsaturated soil, which has promoted the progress of a theoretical model [5–7] and test method [8]. Moreover, there are few studies on the soil-water characteristic curve of complete-intense weathering mudstone, and most of them focus on the water retention performance and pore size distribution of undisturbed soil and remolded soil [9,10], the influence of freeze-thaw cycle on mudstone [11–13], and the expansion characteristics of completely weathered mudstone [14]. It is of great significance to study the soil-water characteristics of complete-intense weathering mudstone for making reasonable prevention and control measures for earth dam seepage, slope strength, and foundation deformation of complete-intense weathering mudstone.

Although the soil-water characteristic curve can predict the strength of the soil, the triaxial shear test as a commonly used indoor test method can more accurately determine the stress-strain relationship and shear strength of the soil. Moreover, the stress and strain in the sample are relatively uniform, the state is clear, and the measurement is simple and reliable in the triaxial shear test. Baoping Wen et al. [15] studied the effect of irrigation water on the shear strength of weathered red mudstone, and found that the erosion of irrigation water led to the decrease of shear strength of weathered red mudstone. Lingwei Kong et al. [16] studied the engineering properties of weathered swelling mudstone. Mudstone has obvious swelling and shrinkage behavior, and the shear strength of expansive samples is significantly lower than that of unsaturated samples. Shuai Zhang et al. [17] studied the influence of rainwater softening on red mudstone and the results showed that the structure of soft rock changes, resulting in strength degradation. Mudstone has a tendency to migrate to soil, and the internal friction angle and cohesion both appear to decrease from fast to slow due to this migration. Some scholars also carried out creep tests on mudstones with different water contents to obtain the strain-time relationship, and analyzed the creep process and creep characteristics to establish a suitable creep model [18–20]. However, the research so far mainly focused on red mudstone or strongly weathered mudstone, and the research on shear strength and deformation characteristics of unsaturated (or saturated) complete-intense weathering mudstone under different frozen temperature conditions is limited.

In practical engineering exploration, mudstone is mostly unsaturated and widely distributed at a certain depth in seasonal frozen areas, such as Northeast China, Indonesia, and even Southeast Asia. Therefore, through indoor geotechnical test, soil-water characteristic test, GDS unsaturated soil triaxial shear test, and GDS temperature-controlled static (dynamic) triaxial shear test, this paper explores the soil-water characteristic curve of unsaturated complete-intense weathering mudstone and the stress-strain relationship and shear strength under different temperature conditions. The research results can provide reference and guidance for the design and construction of practical engineering structures.

## 2 Basic properties of samples

### 2.1 Granulometric composition analysis

**Fig 1:**
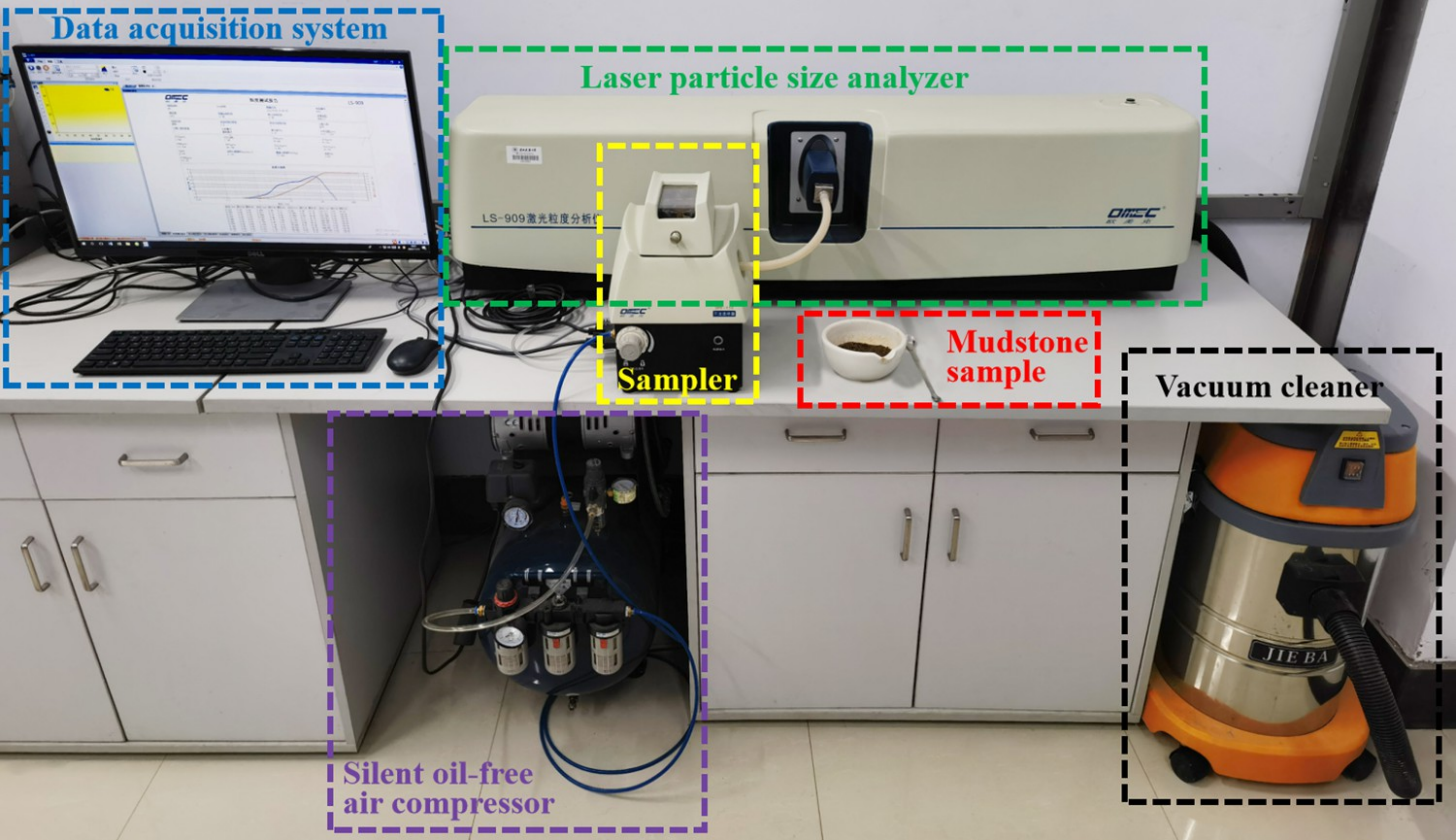
BT-9300H laser particle size analyzer.

**Fig 2:**
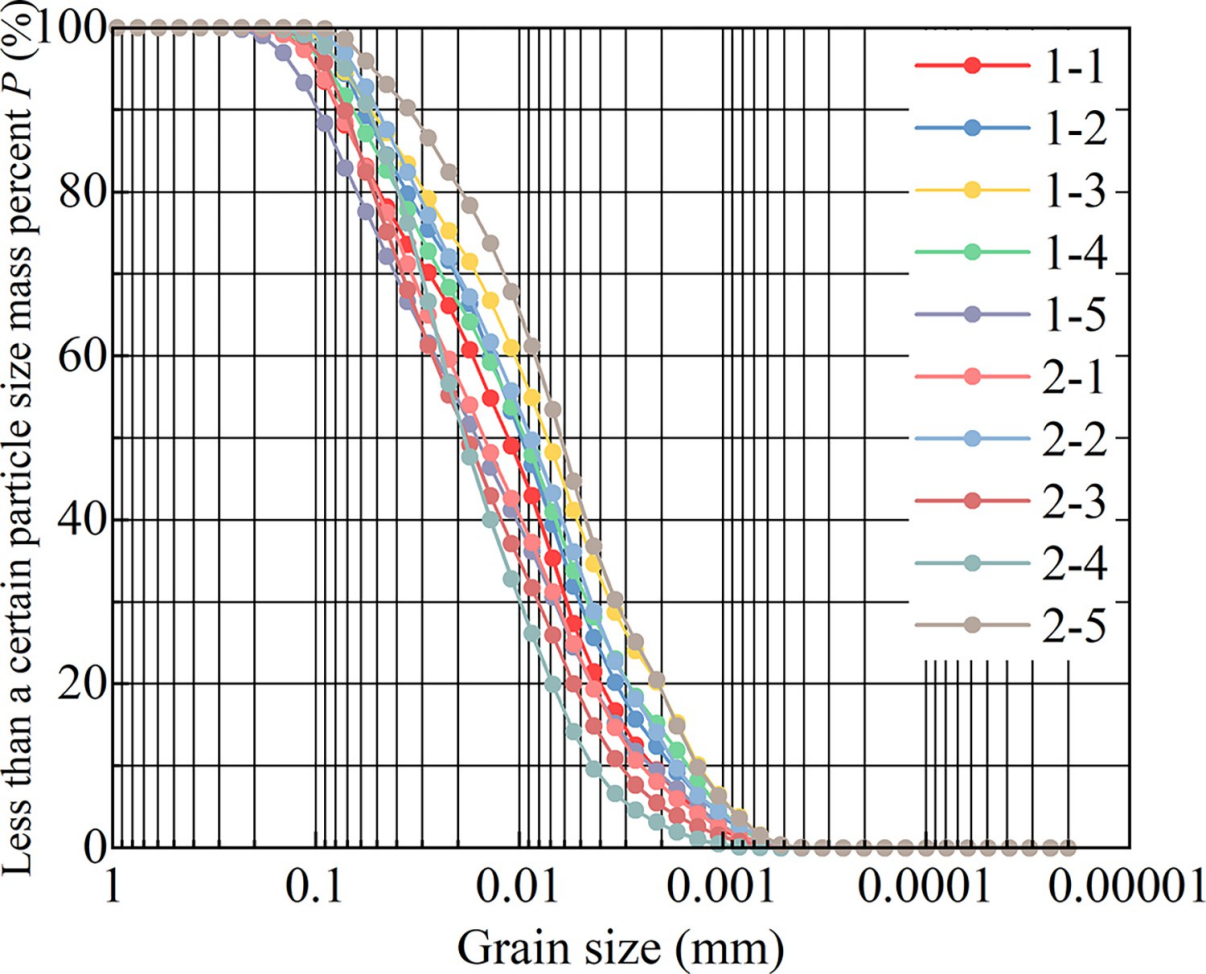
10 groups of complete-intense weathering mudstone particle size gradation.

The complete-intense weathering mudstone used in the sample was taken from the Shuangfeng Station of Urban Rail Transit Line 6 in Changchun City, Jilin Province, China. The freezing depth in winter in Changchun area is about 1.7 m. To avoid the influence of freezing depth on undisturbed samples, the undisturbed complete-intense weathering mudstone with a depth of 8–10 m is selected in this test. Complete-intense weathering mudstone is divided into ten groups. The BT-9300H laser particle size analyzer shown in Fig 1 was used for particle analysis, and the analysis results are shown in Fig 2.

**Fig 3:**
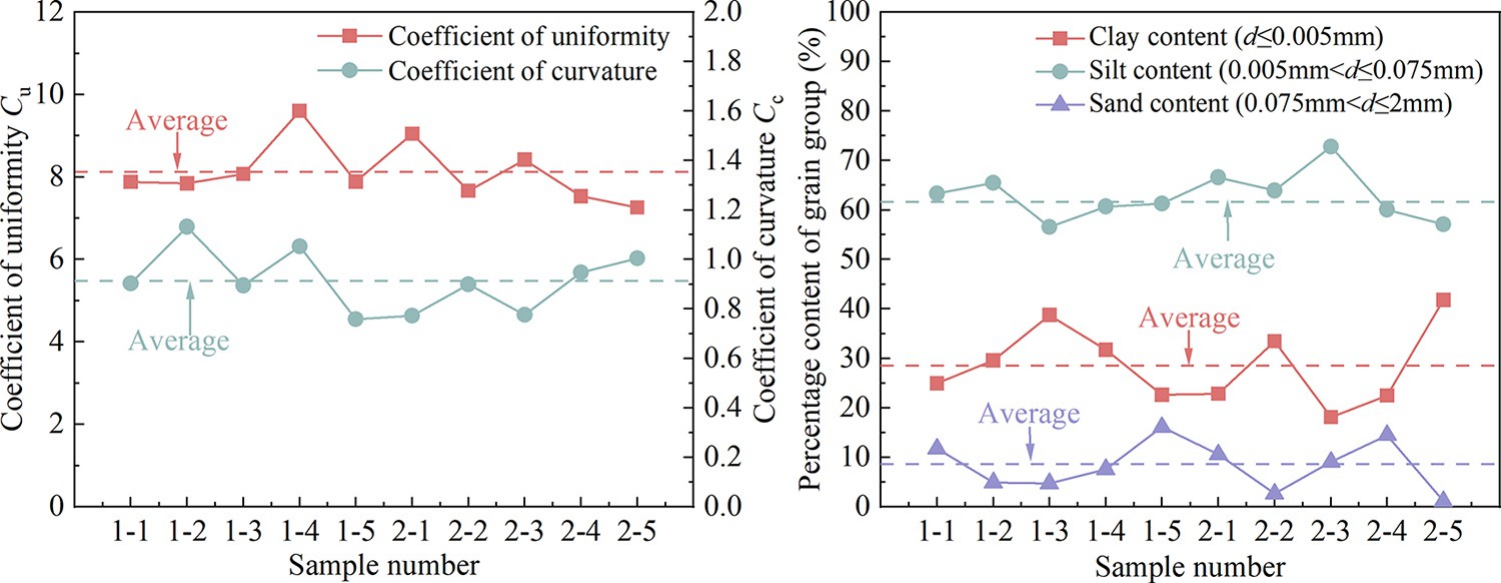
(a) *C*_u_ and *C*. (b) Percentage content of each grain group.

The calculation results of non-uniformity coefficient and curvature coefficient of 10 groups of complete-intense weathering mudstone are shown in Fig 3(a). The percentage of each grain group in the sample is shown in Fig 3(B). The clay content ranged from 18.1 to 41.8%, with an average of 28.63%. The powder content ranged from 56.53 to 72.82%, with an average of 62.78%. The sand content ranged from 1.09 to 16.11%, with an average of 8.29%. The percentage of silt and clay in the complete-intense weathering mudstone of the 10 groups is relatively high, and the sand is less. The mudstone has the characteristics of weak permeability and a significant capillary phenomenon. Therefore, the water migration speed of complete-intense weathering mudstone in unsaturated state is fast and the distance is far when it is subjected to a negative temperature. The temperature has a great influence on the strength and deformation of unsaturated soil.

### 2.2 Basic physical and mechanical properties

**Fig 4:**
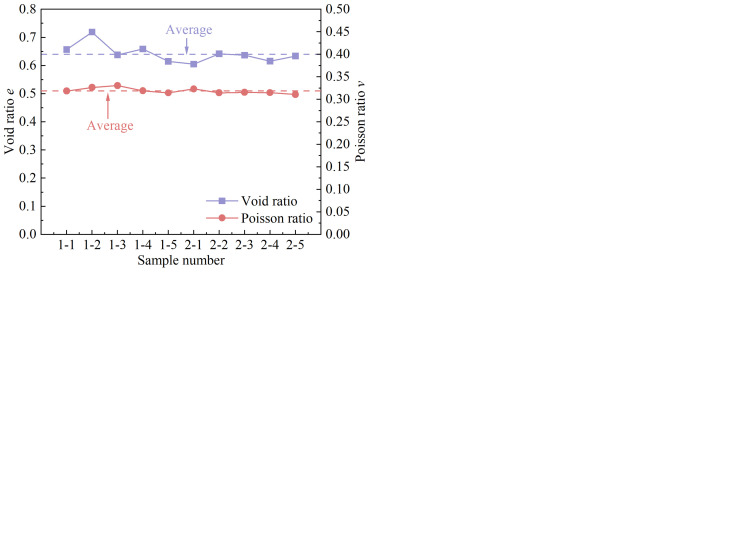
(a) *ρ* and *w*. (b) *w*_L_, *w*_p_, and *I*_p._ (c) *E*_s_ and *a*. (d) *e* and *v*.

Clarifying the basic physical and mechanical properties of the sample plays an important role in analyzing the mechanism of temperature change on the stress-strain curve and shear strength of unsaturated complete-intense weathering mudstone. Therefore, an indoor geotechnical test was carried out to determine the basic physical and mechanical indices of the sample. The variation of physical and mechanical parameters of 10 groups of complete-intense weathering mudstone obtained from the test is shown in Fig 4. The range of variation and the average value are shown in Table 1. Under the action of an external force, the three-phase medium (solid, liquid, and gas) in the complete-intense weathering mudstone will produce displacement, resulting in the decrease of pore volume and compression deformation. The pore gas in unsaturated complete-intense weathering mudstone may swell and shrink due to the change of pressure, which will block the infiltration of groundwater and change the compressibility of soil.

**Table 1:** Range and average of basic physical and mechanical parameters of the specimens.

Physical and mechanical parameters	Range	Average
Natural density *ρ* (g/cm^3^)	1.83~1.96	1.89
Natural water content *w* (%)	17.46~20.75	19.15
Void ratio *e*	0.61~0.72	0.64
Liquid limit *w*_*L*_ (%)	32.43 ~ 47.98	39.48
Plastic limit *w*_p_ (%)	17.66~28.88	23.14
Plasticity index *I*_p_ (%)	13.65 ~ 20.95	16.34
Coefficient of compressibility *a* (MPa^-1^)	0.14~0.19	0.16
Modulus of compression *E*_s_ (MPa)	9.04~11.70	10.48
Poisson’s ratio *v*	0.311~0.331	0.319

## 3 Test scheme

### 3.1 Soil-water characteristic test

**Fig 5:**
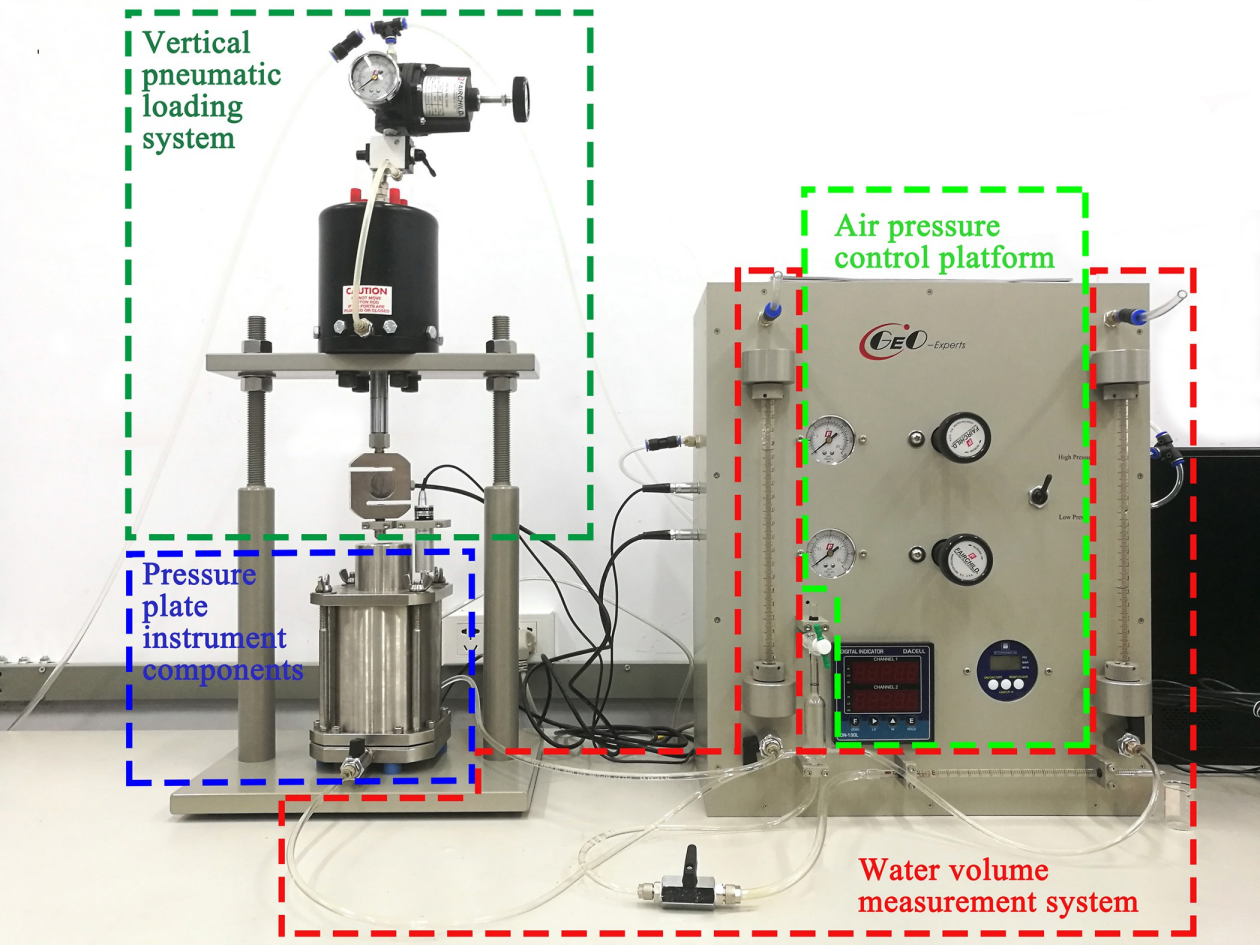
GEO-Experts soil-water characteristic curve pressure plate instrument system.

The commonly used soil-water characteristic curve measurement methods are pressure plate extractor method, tensiometer, and filter paper method. Although the pressure plate extractor method test takes a long time, the test results are more accurate compared to the other methods. As shown in Fig 5, a GEO-Experts soil-water characteristic curve pressure plate instrument system was used to measure the soil-water characteristic curve of undisturbed complete-intense weathering mudstone. The water holding capacity of complete-intense weathering mudstone is studied to determine the matrix suction of fully weathered mudstone in natural state. The test results provide test parameters for the subsequent study of the shear strength of complete-intense weathering mudstone by a GDS unsaturated triaxial apparatus.

The pressure plate instrument test is divided into eight steps: preparation of samples, saturated samples, and clay plates, installation of samples, detection of air tightness of pressure plate instrument components, scouring system bubbles, applying matrix suction, recording the values of drainage and evaporation pipes, and scouring the bottom bubbles of clay plates. The limit air inlet value of the ceramic plate is 500 kPa. When the air inlet value is greater than 500 kPa, the ceramic plate will break down. Considering the maximum safety range of the ceramic plate, the suction test range is 0~450 kPa. The initial suction of the test was 0 kPa, which was gradually increased by 5 kPa every time from 0 to 15 kPa, and then gradually increased by 25 kPa every time to 450kPa, leading to a total of 21 stages. When the matric suction enters a new stage, the water level of the measuring tube is recorded. If the drainage volume of the sample does not change within 24 hours, the interior of the sample can be regarded as reaching the suction balance, and then the matrix suction is changed to start the next stage of the test. After the test, the water content of each stage was calculated by the drainage volume of each stage, and finally the soil-water characteristic curve of the sample was drawn.

### 3.2 Triaxial test of unsaturated complete-intense weathering mudstone

**Fig 6:**
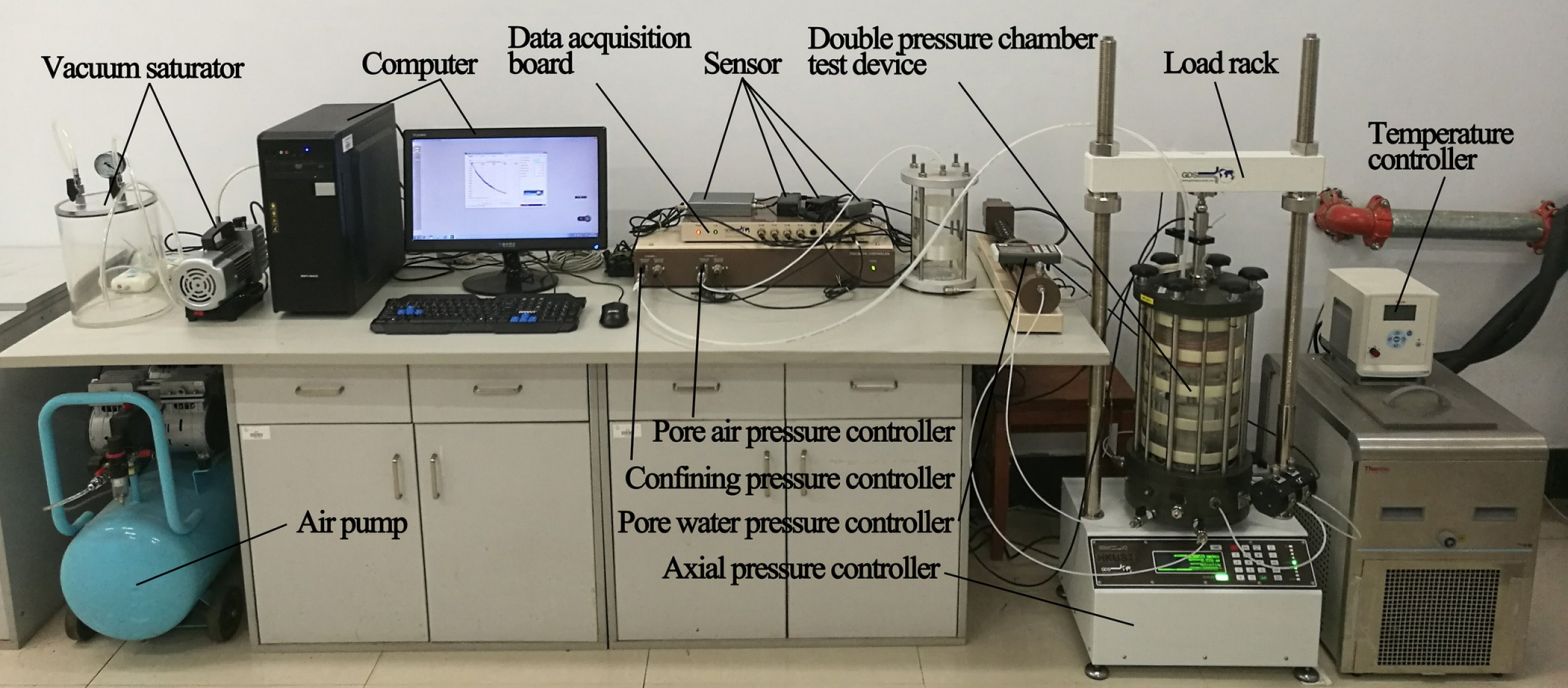
GDS unsaturated soil triaxial test system.

The triaxial test of unsaturated complete-intense weathering mudstone was carried out by using a GDS unsaturated soil triaxial test system, as shown in Fig 6. The undisturbed mudstone was cut into a cylindrical sample of 38×76 mm by a soil cutter, and then vacuum saturated. During the test, the specified matric suction is applied to make the mudstone sample reach a specific unsaturated state, and then the consolidated undrained triaxial shear test is carried out. Based on the soil-water characteristic curve of complete-intense weathering mudstone obtained by pressure plate instrument test, the matrix suction of complete-intense weathering mudstone under natural water content is 210 kPa, so the matrix suction of triaxial test is controlled to 210 kPa. According to the ASTM standard [21] and previous experience, the net confining pressure of the test was controlled to 100, 200, and 300 kPa, respectively, and the shear rate was 0.08%/min. Based on the monthly average temperature of a year in Changchun, the temperature is constant at 20, 0, -5, -10, and -20°C, respectively, and the negative temperature freezing time is set to 12 hours. When the axial deformation reaches 15%, the shear is terminated. There are 15 unsaturated complete-intense weathering mudstone samples. The test parameters at different temperatures are shown in Table 2.

**Table 2:** Triaxial test parameter setting of unsaturated complete-intense weathering mudstone.

Sample number	Matrix suction (kPa)	Temperature (°C)	Net confining pressure (kPa)	Pore gas pressure (kPa)	Pore water pressure (kPa)
1-1-1	210	20	100	215	5
1-1-2			200	215	
1-1-3			300	215	
1-2-1		0	100	215	5
1-2-2			200	215	
1-2-3			300	215	
1-3-1		-5	100	215	5
1-3-2			200	215	
1-3-3			300	215	
1-4-1		-10	100	215	5
1-4-2			200	215	
1-4-3			300	215	
1-5-1		-20	100	215	5
1-5-2			200	215	
1-5-3			300	215	

### 3.3 Triaxial test of saturated fully weathered mudstone

**Fig 7:**
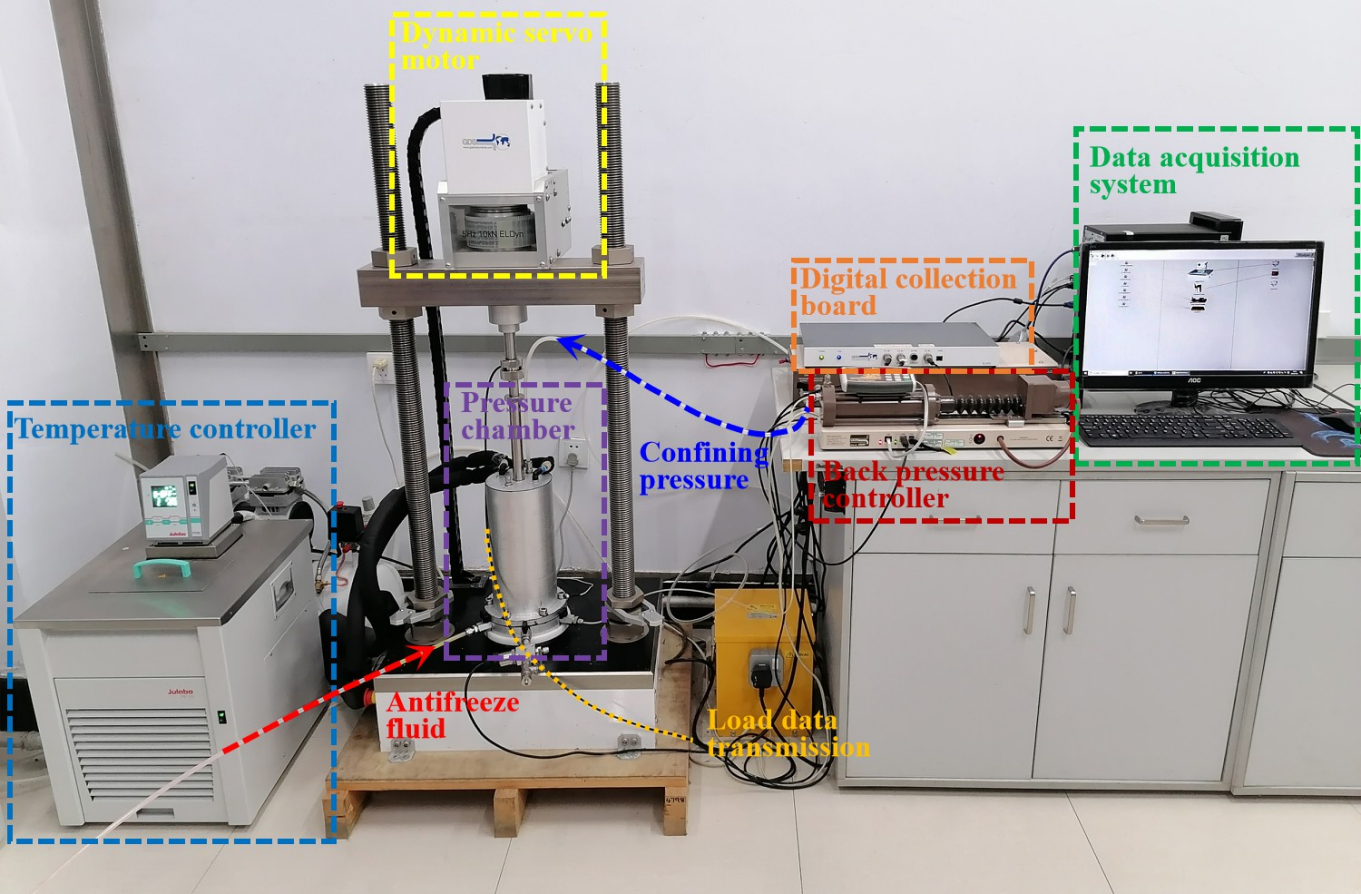
GDS temperature controlled dynamic triaxial test system.

The consolidated undrained triaxial test of saturated completely weathered mudstone is carried out using a GDS temperature controlled static (dynamic) triaxial test system. The test equipment is shown in Fig 7. The equipment can accurately control the temperature of saturated soil samples and realize the freezing of soil samples under constant pressure and the shearing of soil samples at constant temperature, so as to simulate the actual working conditions. To compare with the unsaturated triaxial test data, the temperature was also set to 20, 0, -5, -10, and -20°C, respectively, during the test. The negative temperature freezing time was 12 hours, and the confining pressure was controlled to 100, 200, and 300 kPa, respectively. The test adopts the strain control loading method, the shear rate is 0.1%/min, and the test termination condition is that the strain reaches 15%. There are 15 saturated complete-intense weathering mudstone samples. The design of test parameters under different temperature conditions is shown in Table 3.

**Table 3:** Triaxial test parameters setting of saturated complete-intense weathering mudstone.

Sample number	Matrix suction (kPa)	Temperature (°C)	Net confining pressure (kPa)
2-1-1	0	20	100
2-1-2			200
2-1-3			300
2-2-1		0	100
2-2-2			200
2-2-3			300
2-3-1		-5	100
2-3-2			200
2-3-3			300
2-4-1		-10	100
2-4-2			200
2-4-3			300
2-5-1		-20	100
2-5-2			200
2-5-3			300

## 4 Analysis of soil-water characteristics test results of fully weathered mudstone

**Fig 8:**
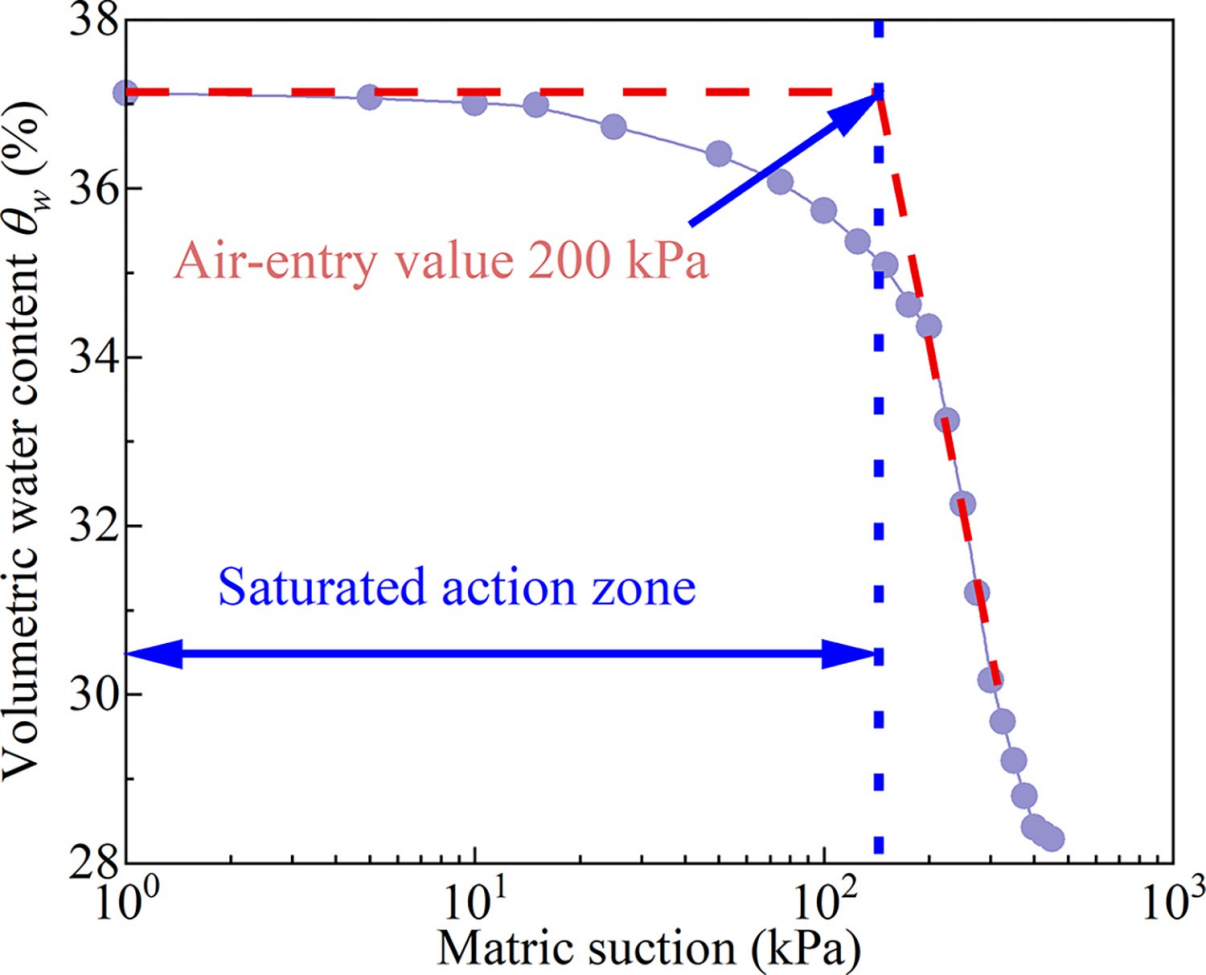
Relationship between volumetric water content and matric suction.

The soil-water characteristic test results of undisturbed complete-intense weathering mudstone using pressure plate instrument are shown in Table 4. The relationship curve between matric suction and volumetric water content is shown in Fig 8. The complete-intense weathering mudstone with a natural water content of 19.15% has a matrix suction of 210 kPa. From Fig 8, it can be seen that when the matrix suction is about 200 kPa, the sample reaches the air-entry value. So, it can be considered that when the matrix suction is between 0 and 200 kPa, the region is the saturation action zone. When the matric suction exceeds 200 kPa, it reaches the second stage of the typical soil-water characteristic curve. The volumetric water content decreases rapidly, and a large amount of gravity water is discharged from the mudstone. The increase of matric suction also enhances the capillary action between soil particles. When the matric suction is 300~450 kPa, the decrease rate of volumetric water content slows down, and the capillary water in the pores of soil particles is gradually discharged. The discharged water finally exists in the form of bound water.

**Table 4:** Soil-water characteristic curve test data.

Matrix suction (kPa)	Displacement volume (ml)	Volumetric water content *θw* (%)	Moisture content *w* (%)	Degree of saturation *S* (%)
0	0 .00	37.13	20.99	100.00
5	0.12	37.08	20.89	99.62
10	0.13	37.01	20.77	99.17
15	0.06	36.99	20.75	99.00
25	0.28	36.73	20.66	98.92
50	0.23	36.41	20.57	98.06
75	0.23	36.08	20.39	97.17
100	0.24	35.74	20.19	96.25
125	0.26	35.37	19.98	95.26
150	0.09	35.09	19.82	94.51
175	0.33	34.62	19.55	93.24
200	0.18	34.36	19.41	92.54
225	0.78	33.25	18.78	89.55
250	0.70	32.26	18.21	86.88
275	0.74	31.21	17.61	84.06
300	0.73	30.17	17.12	81.25
325	0.35	29.68	16.73	79.94
350	0.32	29.22	16.47	78.70
375	0.30	28.80	16.23	77.57
400	0.26	28.43	16.02	76.57
425	0.08	28.35	15.98	76.51
450	0.22	28.29	15.33	75.82

## 5 Effect of temperature on the shear strength of unsaturated complete-intense weathering mudstone

### 5.1 Sample stress-strain analysis

**Fig 9:**
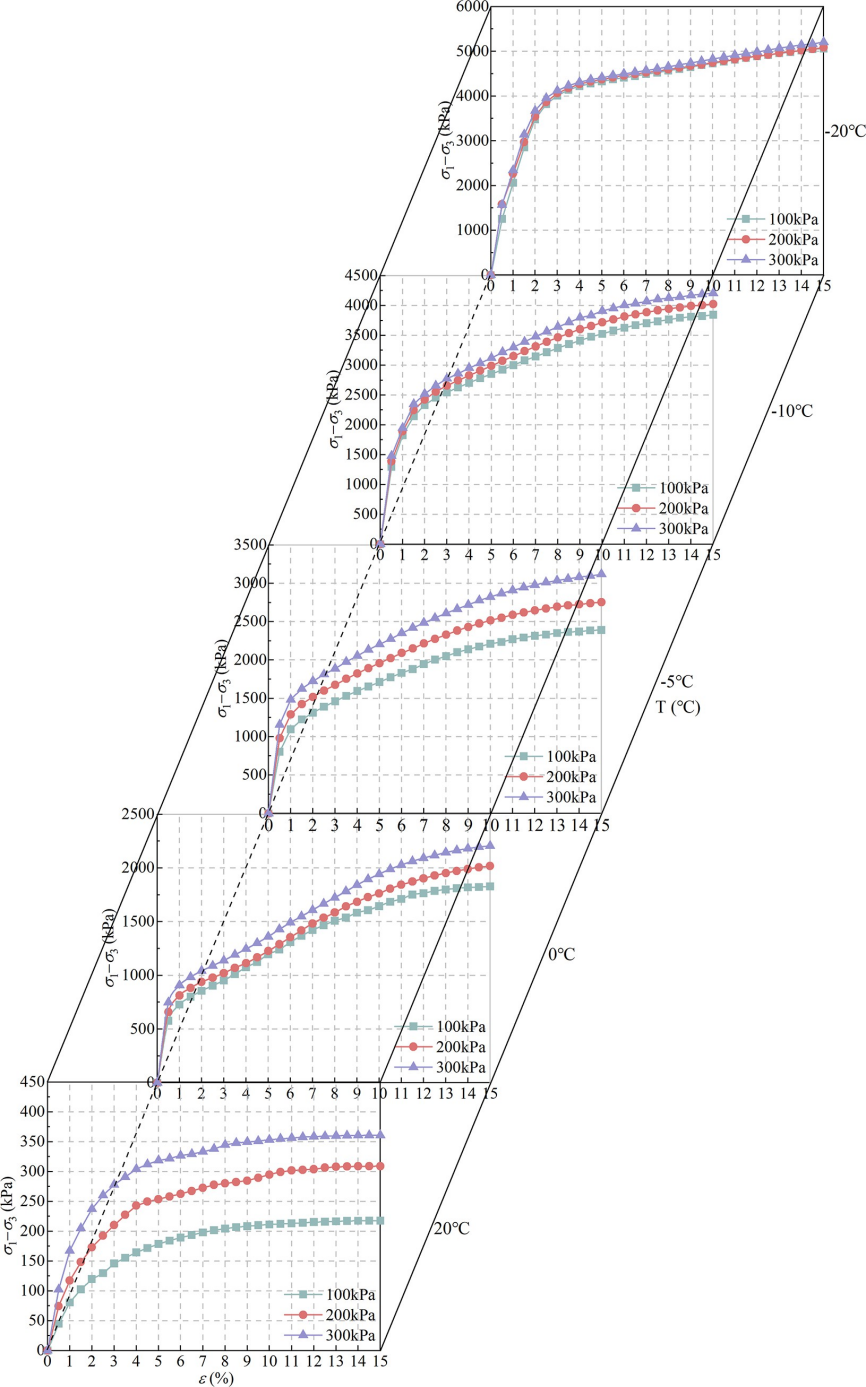
Stress-strain curve of complete-intense weathering mudstone.

The deviatoric stress value is recorded corresponding to every 0.5% of the axial strain in the triaxial shear tests. The stress-strain curves of undisturbed complete-intense weathering mudstone at different temperatures and confining pressures are shown in Fig 9.

The stress-strain relationship is used to reflect the elastic, plastic, yield, and other deformation processes of rock mass under external force, which is the key factor to study the mechanical properties of rock and soil media. In the test samples, the confining pressure changes the shear stress of unsaturated complete-intense weathering mudstone, but has little effect on the overall morphological characteristics of the stress-strain curve. At the same temperature, the change trend of stress-strain curve is basically the same, and the whole is positively correlated. In the early stage of shear, the deviatoric stress (*σ*1-*σ*3) increases rapidly. When the strain increases to 2~4%, the growth rate of deviatoric stress begins to slow down, but the deviatoric stress still keeps increasing. When the confining pressure is constant, the lower the temperature, the faster the stress increase, indicating that the temperature has a great influence on the shear strength of unsaturated complete-intense weathering mudstone. When the temperature is 20°C, the confining pressure increases step by step, the pores between the complete-intense weathering mudstone particles are compressed during the consolidation process, the bonding bite force increases, and the structure becomes denser. So, the shear stress of the sample increases with the increase of confining pressure. Due to the effect of matrix suction, the adsorption strength always exists in the sample, which makes the shear process show certain hardening characteristics. When the temperature decreases from 0 to -20°C, the influence of confining pressure on the rock sample gradually weakens, and the free water inside the unsaturated complete-intense weathering mudstone begins to freeze. The connection between the solid particles changed from the original mudstone-water binary structure to the mudstone-water-ice ternary structure, and finally to the mudstone-ice structure. The change of the connection form improves the stability of the structure, and the mudstone-ice structure can compensate for the loss of soil strength to a certain extent, so the sample has the property of strain hardening during shearing.

### 5.2 Analysis of shear strength parameters of specimens

**Fig 10:**
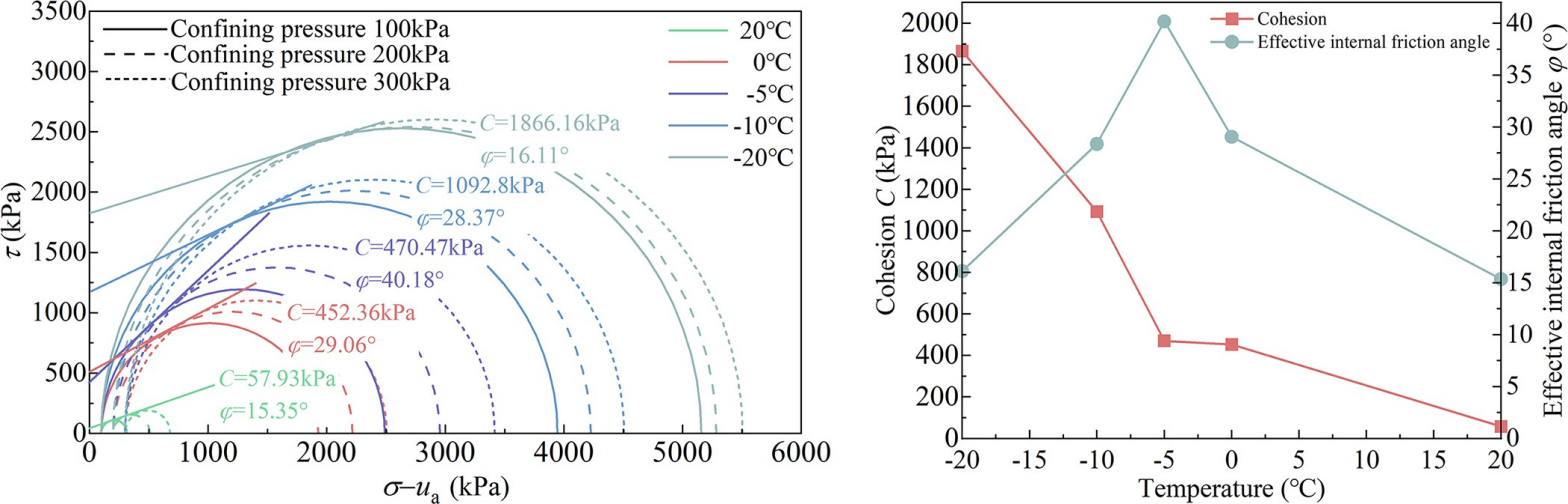
(a) Mohr-Coulomb stress circle and strength envelope. (b) Shear strength parameter.

When the matric suction is 210 kPa, the Mohr-Coulomb stress circle and strength envelope at different temperatures are drawn and are shown in Fig 10(A). The total cohesion *C* and effective internal friction angle *φ* of unsaturated complete-intense weathering mudstone can be obtained, and the results are recorded in Table 5. The curves of total cohesion and effective internal friction angle with temperature are shown in Fig 10(B). Temperature has a great influence on the total cohesion and effective internal friction angle of unsaturated complete-intense weathering mudstone. The total cohesion is formed by the effective cohesion between mudstone particles, the apparent cohesion produced by matrix suction, and the ice cementation cohesion produced by water-ice phase transition. When the temperature is -20°C, the total cohesion reaches the maximum value of 1866.16kPa. When the temperature is 20°C, the minimum total cohesion is 57.93 kPa, and the maximum value is 32.2 times the minimum value. The total cohesion gradually increases with the decrease of temperature. The analysis shows that the lower the temperature, the higher the degree of water-ice phase transition, the mudstone-water structure gradually changes to the mudstone-ice structure, and the increase of ice cementation cohesion in the rock mass increases the total cohesion. The decrease of temperature will also lead to the increase of viscosity of pore water adsorbed on the surface of mudstone particles, which makes the effective to cohesion increase. Therefore, under the same matric suction, the lower the temperature, the greater the total cohesion. To facilitate the analysis, according to the mode of action between particles, the effective internal friction angle of unsaturated complete-intense weathering mudstone is considered to be composed of the suction friction angle under the action of matrix suction, the sliding friction angle of complete-intense weathering mudstone particles, and the static friction angle between closely arranged particles. The matrix suction is a constant, and the effective internal friction angle increases gradually when the temperature decreases from 20 to 0°C. At a positive temperature, with the decrease of temperature, the viscosity of pore water increases, which weakens the lubrication between particles and leads to the increase of sliding friction angle. Therefore, the effective internal friction angle at 0°C is greater than that at 20°C. The adsorption strength always exists in the soil because of the matrix suction, which makes the freezing point of unfrozen water in the pores of the pressurized area decrease during the shearing process. At this time, the critical point of the phase change of water-ice is about -5°C, so 0~-5°C water-ice phase change rate is relatively slow. As the temperature decreases, the percentage of weakly bound water decreases, the percentage of strongly bound water increases, and the sliding friction angle reaches its peak at -5°C when the effective internal friction angle is the largest. When the temperature decreases from -5 to -20°C, the water-ice phase transition in the unsaturated complete-intense weathering mudstone produces fine cracks. The lubrication effect of phase change ice makes the interlocking effect between particles destroyed to varying degrees. The relative sliding between the particles reduces the static friction angle and the sliding friction angle. Therefore, when the temperature is negative, the lower the temperature, the smaller the effective internal friction angle.

**Table 5:** Triaxial test results of unsaturated complete-intense weathering mudstone.

Sample number	Matrix suction (kPa)	Temperature (°C)	Net confining pressure (kPa)	Deviatoric stress (kPa)	Total cohesion *C* (kPa)	Effective angle of internal friction *φ* (°)
1-1-1	210	20	100	217.8	57.93	15.35
1-1-2			200	309.1		
1-1-3			300	360.8		
1-2-1		0	100	1828.2	452.36	29.06
1-2-2			200	2017.8		
1-2-3			300	2205.9		
1-3-1		-5	100	2389.7	470.47	40.18
1-3-2			200	2753.4		
1-3-3			300	3117		
1-4-1		-10	100	3845.5	1092.8	28.37
1-4-2			200	4026.6		
1-4-3			300	4207.7		
1-5-1		-20	100	5058.6	1866.16	16.11
1-5-2			200	5084.5		
1-5-3			300	5206.6		

## 6 Effect of temperature on the shear strength of saturated complete-intense weathering mudstone

### 6.1 Sample stress-strain analysis

**Fig 11:**
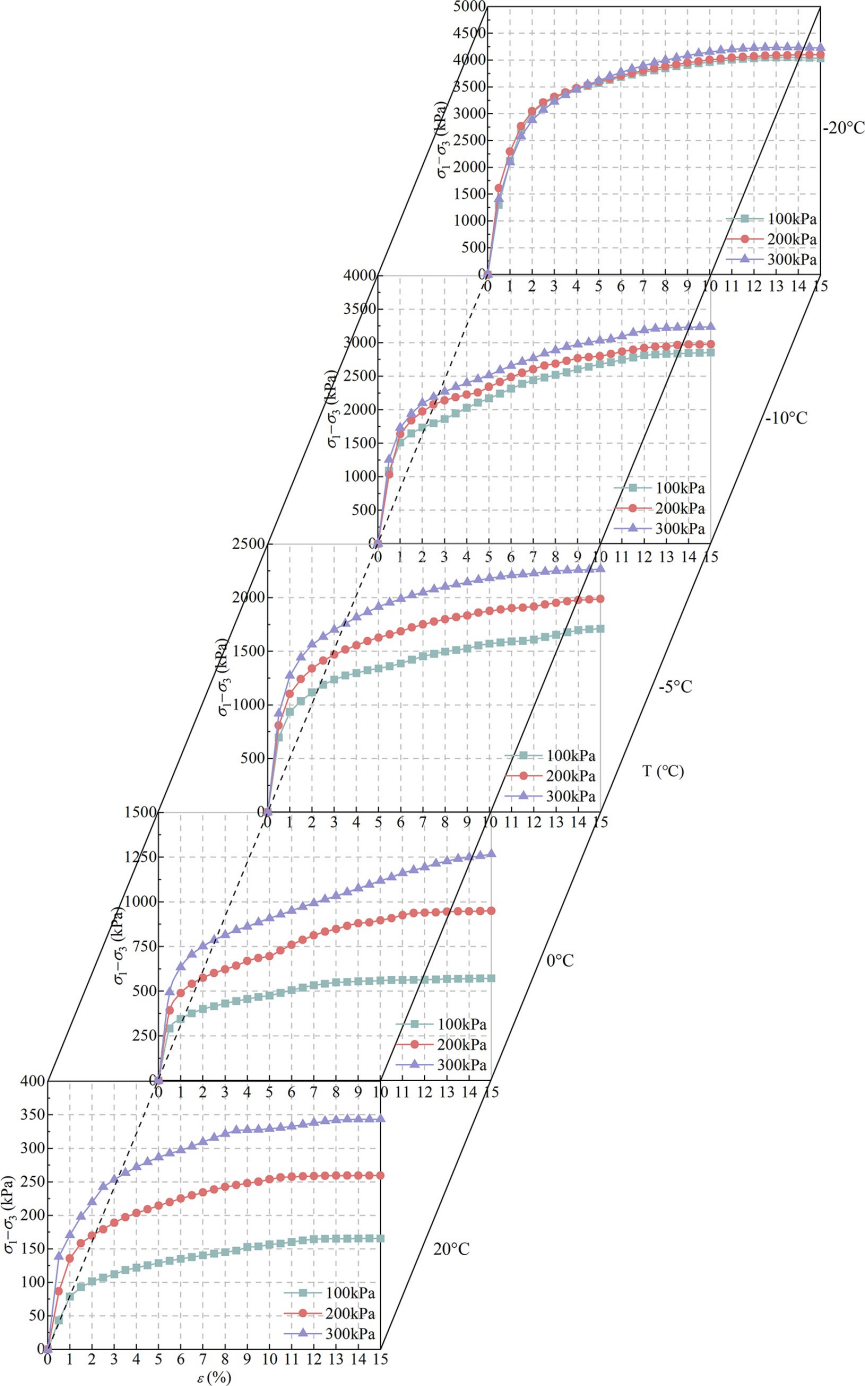
Stress-strain curve of complete-intense weathering mudstone.

The triaxial shear test of saturated complete-intense weathering mudstone at different temperatures was carried out by using a GDS temperature-controlled static (dynamic) triaxial test system. Before the test, the samples were saturated for no less than 48 hours, and the samples were saturated by back pressure during the test. The axial strain is increased by 0.5% every time during the test, and the corresponding deviatoric stress value is recorded. The stress-strain curves of saturated fully weathered mudstone at different temperatures and confining pressures are shown in Fig 11.

It can be seen from the figure that the lower the temperature, the weaker the influence of confining pressure on the deviatoric stress. At -20°C, the confining pressure has little effect on the deviatoric stress. With the increase of temperature, the effect of confining pressure on the deviatoric stress is more significant. At 20°C, the deviatoric stress corresponding to the confining pressure of 300 kPa tends to be stable and is twice the stable value of the deviatoric stress corresponding to 100 kPa. The confining pressure changes the shear stress of the complete-intense weathering mudstone, which has little effect on the overall morphological characteristics of the stress-strain curve. The lower the temperature, the higher the degree of water-ice phase change, and the strength of the rock mass is improved. At this time, the confining pressure of 300 kPa is too small compared with the axial pressure at the time of failure, so the change of stress-strain curve is very small. Under the same confining pressure, the lower the temperature, the faster the growth rate of deviatoric stress, indicating that the temperature has a great influence on the shear strength of saturated complete-intense weathering mudstone. When the temperature is 25°C, with the increase of confining pressure, the mudstone particles become more compact during the consolidation process of the sample, and the shear stress of the sample increases with the increase of confining pressure. With the decrease of temperature, the free water in the saturated complete-intense weathering mudstone sample began to freeze, and the cementation state between mudstone particles changed from the original mudstone-water gel connection to mudstone-ice connection, which improved the structure of mudstone. When the temperature is reduced to -20°C, the mudstone-ice bonding strength can meet the loss of rock strength, so the saturated fully weathered mudstone has the characteristics of strain hardening during shearing.

### 6.2 Analysis of shear strength parameters of saturated complete-intense weathering mudstone

**Fig 12:**
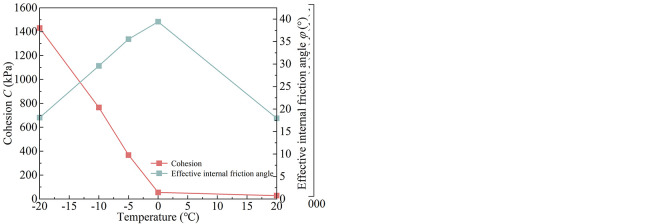
(a) Mohr-Coulomb stress circle and strength envelope. (b) Shear strength parameter.

The shear strength parameters of saturated complete-intense weathering mudstone (matrix suction is 0) obtained from the test are shown in Table 6. The Mohr-Coulomb stress circle and strength envelope at different temperatures are shown in Fig 12(A). The curves of total cohesion and effective internal friction angle with temperature are shown in Fig 12(B).

**Table 6:** Triaxial test results of saturated complete-intense weathering mudstone.

Sample number	Matrix suction (kPa)	Temperature (°C)	Net confining pressure (kPa)	Deviatoric stress (kPa)	Total cohesion *C* (kPa)	Effective angle of internal friction *φ* (°)
2-1-1	0	20	100	165.5	28.39	17.95
2-1-2			200	259.6		
2-1-3			300	343.6		
2-2-1		0	100	572.8	55.38	39.42
2-2-2			200	950.6		
2-2-3			300	1267.6		
2-3-1		-5	100	1710.3	368.42	35.56
2-3-2			200	1988.2		
2-3-3			300	2266.1		
2-4-1		-10	100	2854	765.97	29.61
2-4-2			200	2978.6		
2-4-3			300	3237		
2-5-1		-20	100	4053	1431.53	18.11
2-5-2			200	4103.3		
2-5-3			300	4230.7		

**Fig 13:**
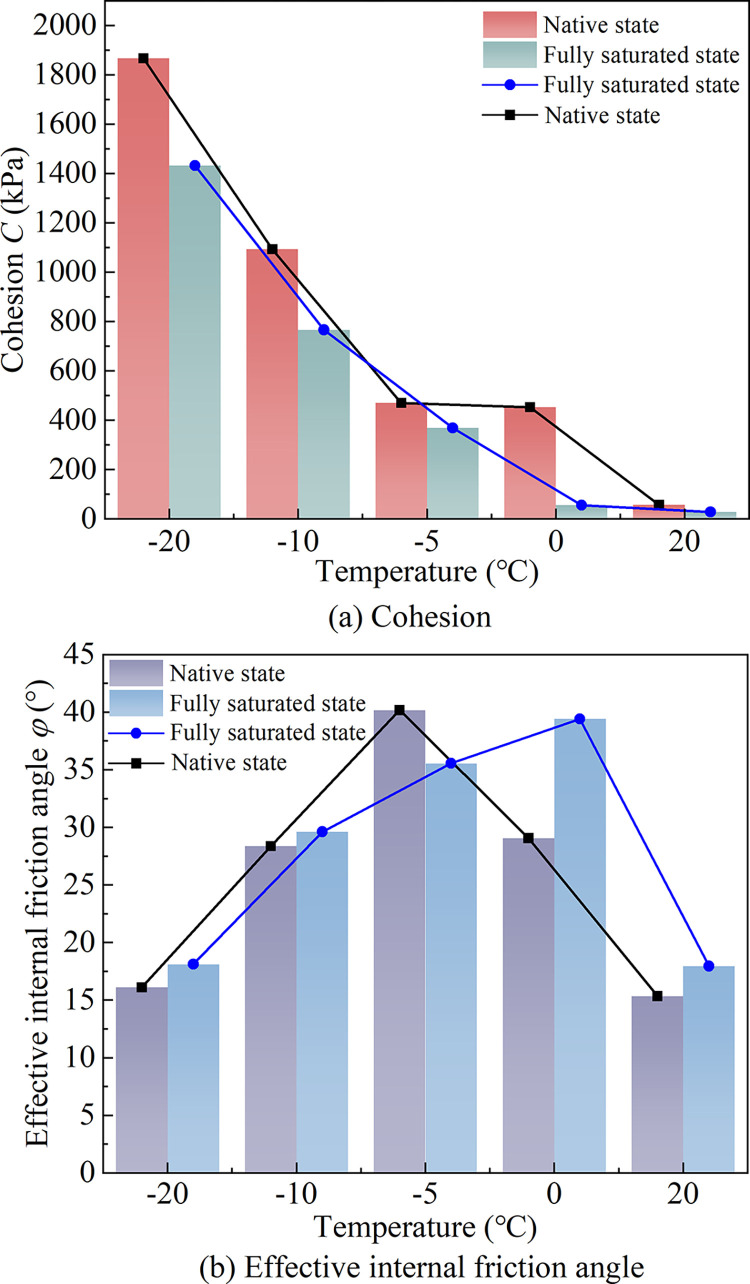
Comparison of shear strength parameters.

The comparison of shear strength parameters of saturated and unsaturated complete-intense weathering mudstone at different temperatures is shown in Fig 13. From the figure, it can be seen that under different temperature conditions, the cohesion of unsaturated complete-intense weathering mudstone with natural water content (with a matrix suction of 210 kPa) is greater than that of saturated complete-intense weathering mudstone at the same temperature. There are differences in values but the change trend is basically the same. Compared with unsaturated complete-intense weathering mudstone, the test of shear strength parameters of saturated complete-intense weathering mudstone is simpler and the results are more conservative. In practical engineering, the basic properties of unsaturated complete-intense weathering mudstone can be predicted by testing the shear strength parameters of saturated complete-intense weathering mudstone.

## 7 Conclusions

Based on indoor geotechnical, pressure plate, and triaxial shear tests, the strength and deformation characteristics of complete-intense weathering mudstone under natural water content state and saturated state are studied and analyzed. The following conclusions are drawn from this study:

1. The average content of clay, silt and sand in complete-intense weathering mudstone is 28.63, 62.78, and 8.29%, respectively. The percentage of silt and clay is relatively high, and the sand is less. The mudstone has the characteristics of weak permeability and a significant capillary phenomenon. When the complete-intense weathering mudstone in unsaturated state is subjected to a negative temperature, the water migration is fast and the distance is far. The temperature has a great influence on the strength and deformation of unsaturated complete-intense weathering mudstone.
2. The complete-intense weathering mudstone with a natural water content of 19.15% has a matrix suction of 210 kPa. When the matric suction is less than 200 kPa, the complete-intense weathering mudstone is saturated. When the matric suction exceeds 200 kPa, the volumetric water content of the mudstone decreases rapidly, and a large amount of gravity water is discharged from the mudstone. The increase of matric suction also enhances the capillary action between soil particles.
3. At the same temperature, the change trend of stress-strain curve is basically the same, and the whole is positively correlated. In the early stage of shear, the deviatoric stress (*σ*1-*σ*3) increases rapidly. When the strain increases to 2~4%, the growth rate of deviatoric stress begins to slow down, but the deviatoric stress still keeps increasing. At the same temperature, the shear stress of the sample increases with the increase of confining pressure. When the temperature decreases from 0 to -20°C, the influence of confining pressure on samples gradually weakens. The solid particles tend to be bonded by ice cementation from the original water cementation, and the change of the connection form improves the stability of the structure. So, the complete-intense weathering mudstone has the property of strain hardening during shearing.
4. The total cohesion is formed by the effective cohesion between mudstone particles, the apparent cohesion produced by matrix suction, and the ice cementation cohesion produced by water-ice phase transition. Under the same matric suction, the total cohesion increases with the decrease of temperature. The effective internal friction angle of unsaturated completely weathered mudstone is composed of the suction friction angle under the action of matric suction, the sliding friction angle between particles, and the static friction angle between closely arranged particles. At a positive temperature, the effective friction angle increases with the decrease of temperature. When the temperature is negative, the lower the temperature, the smaller the effective internal friction angle.
5. Under different temperature conditions, the cohesion of unsaturated complete-intense weathering mudstone is greater than that of saturated complete-intense weathering mudstone at the same temperature. There are only numerical differences between the two cohesions, and their trend is basically the same. Compared with unsaturated complete-intense weathering mudstone, the test of shear strength parameters of saturated complete-intense weathering mudstone is relatively simple and conservative. In practical engineering, the basic properties of unsaturated complete-intense weathering mudstone can be predicted by testing the shear strength parameters of saturated complete-intense weathering mudstone.

## Supporting information

S1 Raw data (XLSX)
